# Supplementary material for: Gut Microbiota and Obsessive–Compulsive Disorder: A Systematic Review of Mechanistic Links, Evidence from Human and Preclinical Studies, and Therapeutic Prospects
Source: Life (Basel). 2025 Oct 10;15(10):1585. doi: 10.3390/life15101585 (PMC12565408; doi:10.3390/life15101585)
Supplement: Supplementary file 1 [file life-15-01585-s001.zip › Supplementary Table S1.pdf]

Supplementary Table S1. Detailed search strategies used in the systematic review.

Step	Database	Search string
**Step 1** (OCD + immune/metabolic/epigenetic/neurotransmitter terms)	**EMBASE**	('obsessive compulsive disorder'/exp OR 'obsessive compulsive disorder':ti,ab,kw OR 'OCD':ti,ab,kw) AND ('cytokine'/exp OR cytokine*:ti,ab,kw OR 'chemokine'/exp OR chemokine*:ti,ab,kw OR 'interleukin'/exp OR interleukin*:ti,ab,kw OR 'interleukin 1 beta':ti,ab,kw OR 'IL-1beta':ti,ab,kw OR 'IL-6':ti,ab,kw OR 'IL-10':ti,ab,kw OR 'IL-17':ti,ab,kw OR 'IL-22':ti,ab,kw OR 'TNF':ti,ab,kw OR 'NF-kB':ti,ab,kw OR 'toll like receptor'/exp OR 'TLR':ti,ab,kw OR 'toll-like receptor':ti,ab,kw OR 'microglia'/exp OR microglia:ti,ab,kw OR 'macrophage'/exp OR macrophage*:ti,ab,kw OR 'short chain fatty acid'/exp OR 'SCFA':ti,ab,kw OR 'short chain fatty acid':ti,ab,kw OR butyrate:ti,ab,kw OR acetate:ti,ab,kw OR propionate:ti,ab,kw OR mitochondria*/exp OR mitochondria*:ti,ab,kw OR glycolysis/exp OR glycolysis:ti,ab,kw OR 'oxidative phosphorylation':ti,ab,kw OR 'energy metabolism':ti,ab,kw OR epigenet*:ti,ab,kw OR methylation:ti,ab,kw OR acetylation:ti,ab,kw OR histone/exp OR histone:ti,ab,kw OR 'histone methylation':ti,ab,kw OR 'histone acetylation':ti,ab,kw

		OR chromatin:ti,ab,kw OR 'chromatin remodeling':ti,ab,kw OR miRNA:ti,ab,kw OR lncRNA:ti,ab,kw OR ncRNA:ti,ab,kw OR 'gene expression':ti,ab,kw OR ghrelin:ti,ab,kw OR 'cholecystokinin'/exp OR CCK:ti,ab,kw OR 'glucagon like peptide 1'/exp OR GLP-1:ti,ab,kw OR 'peptide YY'/exp OR PYY:ti,ab,kw OR leptin:ti,ab,kw OR cortisol:ti,ab,kw OR 'adrenocorticotropic hormone'/exp OR ACTH:ti,ab,kw OR 'corticotropin releasing hormone'/exp OR CRH:ti,ab,kw OR neuropeptide*:ti,ab,kw OR hypothalamus/exp OR hypothalamus:ti,ab,kw OR serotonin/exp OR serotonin:ti,ab,kw OR dopamine/exp OR dopamine:ti,ab,kw OR GABA:ti,ab,kw OR glutamate:ti,ab,kw)
	**PubMed**	("Obsessive-Compulsive Disorder"[Mesh] OR "obsessive compulsive disorder"[tiab] OR "obsessive-compulsive disorder"[tiab] OR OCD[tiab]) AND ("Cytokines"[Mesh] OR cytokine*[tiab] OR "Chemokines"[Mesh] OR chemokine*[tiab] OR "Interleukins"[Mesh] OR interleukin*[tiab] OR "interleukin 1 beta"[tiab] OR "IL-1beta"[tiab] OR "IL-6"[tiab] OR "IL-10"[tiab] OR "IL-17"[tiab] OR "IL-22"[tiab]

		OR "Tumor Necrosis Factor-alpha"[Mesh] OR TNF[tiab] OR "NF-kB"[tiab] OR "Toll-Like Receptors"[Mesh] OR TLR[tiab] OR "toll like receptor"[tiab] OR "toll-like receptor"[tiab] OR "Microglia"[Mesh] OR microglia[tiab] OR "Macrophages"[Mesh] OR macrophage*[tiab] OR "short chain fatty acid"[tiab] OR SCFA[tiab] OR "short chain fatty acids"[tiab] OR butyrate[tiab] OR acetate[tiab] OR propionate[tiab] OR mitochondria*[tiab] OR "Glycolysis"[Mesh] OR glycolysis[tiab] OR "Oxidative Phosphorylation"[Mesh] OR "oxidative phosphorylation"[tiab] OR "Energy Metabolism"[Mesh] OR "energy metabolism"[tiab] OR epigenet*[tiab] OR methylation[tiab] OR acetylation[tiab] OR "Histones"[Mesh] OR histone[tiab] OR "histone methylation"[tiab] OR "histone acetylation"[tiab] OR chromatin[tiab] OR "chromatin remodeling"[tiab] OR miRNA[tiab] OR lncRNA[tiab] OR ncRNA[tiab] OR "gene expression"[tiab] OR "Ghrelin"[Mesh] OR ghrelin[tiab] OR "Cholecystokinin"[Mesh] OR CCK[tiab] OR "Glucagon-Like Peptide 1"[Mesh] OR GLP-1[tiab] OR "Peptide YY"[Mesh] OR PYY[tiab] OR "Leptin"[Mesh] OR leptin[tiab] OR

		"Hydrocortisone"[Mesh] OR cortisol[tiab]<br>OR "Adrenocorticotropic Hormone"[Mesh] OR ACTH[tiab]<br>OR "Corticotropin-Releasing Hormone"[Mesh] OR CRH[tiab] OR neuropeptide*[tiab] OR "Hypothalamus"[Mesh] OR hypothalamus[tiab] OR "Serotonin"[Mesh] OR serotonin[tiab]<br>OR "Dopamine"[Mesh] OR dopamine[tiab] OR "gamma-Aminobutyric Acid"[Mesh] OR GABA[tiab]<br>OR "Glutamic Acid"[Mesh] OR glutamate[tiab])
	**PsychInfo**	((obsessive compulsive disorder OR obsessive-compulsive disorder OR OCD).ti,ab.)<br>AND<br>(cytokine*.ti,ab. OR chemokine*.ti,ab. OR interleukin*.ti,ab. OR "interleukin 1 beta".ti,ab. OR "IL-1beta".ti,ab. OR "IL-6".ti,ab. OR "IL-10".ti,ab. OR "IL-17".ti,ab. OR "IL-22".ti,ab. OR TNF.ti,ab. OR "NF-kB".ti,ab. OR TLR.ti,ab. OR "toll like receptor".ti,ab. OR "toll-like receptor".ti,ab. OR microglia.ti,ab. OR macrophage*.ti,ab. OR "short chain fatty acid".ti,ab. OR SCFA.ti,ab. OR "short chain fatty acids".ti,ab. OR butyrate.ti,ab. OR acetate.ti,ab. OR propionate.ti,ab. OR mitochondria*.ti,ab. OR glycolysis.ti,ab. OR "oxidative phosphorylation".ti,ab. OR "energy metabolism".ti,ab.

		OR epigenet*.ti,ab. OR methylation.ti,ab. OR acetylation.ti,ab. OR histone.ti,ab. OR "histone methylation".ti,ab. OR "histone acetylation".ti,ab. OR chromatin.ti,ab. OR "chromatin remodeling".ti,ab. OR miRNA.ti,ab. OR lncRNA.ti,ab. OR ncRNA.ti,ab. OR "gene expression".ti,ab. OR ghrelin.ti,ab. OR CCK.ti,ab. OR "glucagon like peptide 1".ti,ab. OR GLP-1.ti,ab. OR "peptide YY".ti,ab. OR PYY.ti,ab. OR leptin.ti,ab. OR cortisol.ti,ab. OR ACTH.ti,ab. OR "corticotropin releasing hormone".ti,ab. OR CRH.ti,ab. OR neuropeptide*.ti,ab. OR hypothalamus.ti,ab. OR serotonin.ti,ab.<br>OR dopamine.ti,ab. OR GABA.ti,ab. OR glutamate.ti,ab.)
	**Web of Science**	TS=("obsessive compulsive disorder" OR "obsessive-compulsive disorder" OR OCD)<br>AND<br>TS=(cytokine* OR chemokine* OR interleukin* OR "interleukin 1 beta" OR "IL-1beta"<br>OR "IL-6" OR "IL-10" OR "IL-17" OR "IL-22" OR TNF OR "NF-kB" OR TLR OR "toll like receptor" OR "toll-like receptor" OR microglia OR macrophage*<br>OR "short chain fatty acid" OR SCFA OR "short chain fatty acids" OR butyrate OR acetate OR propionate OR mitochondria* OR glycolysis

		OR "oxidative phosphorylation" OR "energy metabolism" OR epigenet* OR methylation OR acetylation OR histone OR "histone methylation" OR "histone acetylation"<br>OR chromatin OR "chromatin remodeling" OR miRNA OR lncRNA OR ncRNA OR "gene expression" OR ghrelin OR CCK OR "glucagon like peptide 1" OR GLP-1 OR "peptide YY" OR PYY OR leptin OR cortisol<br>OR ACTH OR "corticotropin releasing hormone" OR CRH OR neuropeptide* OR hypothalamus OR serotonin OR dopamine OR GABA OR glutamate)
	**Chocrane library**	(MeSH descriptor: [Obsessive-Compulsive Disorder] explode all trees<br>OR "obsessive compulsive disorder":ti,ab,kw OR "obsessive-compulsive disorder":ti,ab,kw OR OCD:ti,ab,kw)<br>AND<br>(MeSH descriptor: [Cytokines] explode all trees OR cytokine*:ti,ab,kw<br>OR MeSH descriptor: [Chemokines] explode all trees OR chemokine*:ti,ab,kw<br>OR MeSH descriptor: [Interleukins] explode all trees OR interleukin*:ti,ab,kw<br>OR "interleukin 1 beta":ti,ab,kw OR "IL-1beta":ti,ab,kw OR "IL-6":ti,ab,kw OR "IL-10":ti,ab,kw OR "IL-17":ti,ab,kw OR "IL-22":ti,ab,kw OR MeSH

		descriptor: [Tumor Necrosis Factor-alpha] explode all trees OR TNF:ti,ab,kw OR "NF-kB":ti,ab,kw OR MeSH descriptor: [Toll-Like Receptors] explode all trees OR TLR:ti,ab,kw OR "toll like receptor":ti,ab,kw OR "toll-like receptor":ti,ab,kw OR MeSH descriptor: [Microglia] explode all trees OR microglia:ti,ab,kw OR MeSH descriptor: [Macrophages] explode all trees OR macrophage*:ti,ab,kw OR "short chain fatty acid":ti,ab,kw OR SCFA:ti,ab,kw OR "short chain fatty acids":ti,ab,kw OR butyrate:ti,ab,kw OR acetate:ti,ab,kw OR propionate:ti,ab,kw OR mitochondria*:ti,ab,kw OR MeSH descriptor: [Glycolysis] explode all trees OR glycolysis:ti,ab,kw OR MeSH descriptor: [Oxidative Phosphorylation] explode all trees OR "oxidative phosphorylation":ti,ab,kw OR MeSH descriptor: [Energy Metabolism] explode all trees OR "energy metabolism":ti,ab,kw OR epigenet*:ti,ab,kw OR methylation:ti,ab,kw OR acetylation:ti,ab,kw OR MeSH descriptor: [Histones] explode all trees OR histone:ti,ab,kw OR "histone methylation":ti,ab,kw OR "histone acetylation":ti,ab,kw OR chromatin:ti,ab,kw OR "chromatin remodeling":ti,ab,kw OR miRNA:ti,ab,kw OR lncRNA:ti,ab,kw OR

		ncRNA:ti,ab,kw OR "gene expression":ti,ab,kw OR MeSH descriptor: [Ghrelin] explode all trees OR ghrelin:ti,ab,kw OR MeSH descriptor: [Cholecystokinin] explode all trees OR CCK:ti,ab,kw OR MeSH descriptor: [Glucagon-Like Peptide 1] explode all trees OR GLP-1:ti,ab,kw OR MeSH descriptor: [Peptide YY] explode all trees OR PYY:ti,ab,kw OR MeSH descriptor: [Leptin] explode all trees OR leptin:ti,ab,kw OR MeSH descriptor: [Hydrocortisone] explode all trees OR cortisol:ti,ab,kw OR MeSH descriptor: [Adrenocorticotropic Hormone] explode all trees OR ACTH:ti,ab,kw OR MeSH descriptor: [Corticotropin-Releasing Hormone] explode all trees OR CRH:ti,ab,kw OR neuropeptide*:ti,ab,kw OR MeSH descriptor: [Hypothalamus] explode all trees OR hypothalamus:ti,ab,kw OR MeSH descriptor: [Serotonin] explode all trees OR serotonin:ti,ab,kw OR MeSH descriptor: [Dopamine] explode all trees OR dopamine:ti,ab,kw OR MeSH descriptor: [gamma-Aminobutyric Acid] explode all trees OR GABA:ti,ab,kw OR MeSH descriptor: [Glutamic Acid] explode all trees OR glutamate:ti,ab,kw)
**Step 2** (OCD + microbiota terms)	**EMBASE**	(‘obsessive compulsive disorder’/exp OR

		OCD:ti,ab,kw) AND (‘gut microbiota’/exp OR microbiome:ti,ab,kw OR ‘gut-brain axis’:ti,ab,kw OR ‘intestinal permeability’:ti,ab,kw OR FMT:ti,ab,kw OR “fecal microbiota transplant”:ti,ab,kw)
	**PubMed**	(“Obsessive-Compulsive Disorder”[Mesh] OR OCD[tiab]) AND (“gut microbiota”[Mesh] OR microbiome[tiab] OR “gut-brain axis”[tiab] OR “intestinal permeability”[tiab] OR FMT[tiab] OR “fecal microbiota transplant”[tiab])
	**PsycINFO**	(OCD OR “obsessive-compulsive disorder”) AND (microbiome OR “gut microbiota” OR “gut-brain axis” OR “intestinal permeability” OR FMT OR “fecal microbiota transplant”)
	**Web of Science**	TS = (“obsessive-compulsive disorder” OR OCD) AND TS = (“gut microbiota” OR microbiome OR “gut-brain axis” OR “intestinal permeability” OR FMT OR “fecal microbiota transplant”)
	**Cochrane library**	#1 MeSH descriptor: [Obsessive-Compulsive Disorder] explode all trees<br>#2 ("obsessive compulsive disorder" OR "obsessive-compulsive disorder" OR OCD):ti,ab,kw<br>#3 (obsess* NEAR/3 compuls*):ti,ab,kw<br>#4: #1 OR #2 OR #3<br><br>#5 MeSH descriptor: [Microbiota] explode all trees

		#6 MeSH descriptor: [Gastrointestinal Microbiome] explode all trees<br>#7 MeSH descriptor: [Fecal Microbiota Transplantation] explode all trees<br>#8 MeSH descriptor: [Intestinal Permeability] explode all trees<br>#9 (microbiot* OR microbiome* OR microflora*):ti,ab,kw<br>#10 ((gut OR intestin* OR gastrointestin* OR fecal OR faecal OR stool*) NEAR/3 (microbiot* OR microbiome* OR microflora*)):ti,ab,kw<br>#11 (FMT OR ((fecal OR faecal OR stool*) NEAR/3 (microbiota OR microbiome OR microflora OR transplant* OR bacteriotherap* OR engraft*))):ti,ab,kw<br>#12 ("gut-brain axis" OR ((gut OR intestin* OR microbio*) NEAR/3 brain)):ti,ab,kw<br>#13 ("intestinal permeability" OR "leaky gut" OR (gut NEAR/3 permeability) OR (intestinal NEAR/3 barrier)):ti,ab,kw<br>#14 #5 OR #6 OR #7 OR #8 OR #9 OR #10 OR #11 OR #12 OR #13<br><br>#15: #4 AND #14
